# St. Thomas's Hospital Medical School

**Published:** 1894-06-16

**Authors:** 


### ST. THOMAS'S HOSPITAL MEDICAL SCHOOL.

ON Saturday afternoon last an interesting and important ceremony took place at St. Thomas's Hospital. As President of the hospital the Duke of Connaught had the satisfaction of declaring open two handsome blocks of buildings which, with an older and original part, go to make up three sides of a square at the Lambeth Palace end of the hospital grounds. St. Thomas's Hospital has by necessity been sadly behind most of the London teaching hospitals in its accommodation for students, both as regards its laboratory arrangements and the comforts of its students' club.

The opening ceremony, which took place in the open-air, was not favoured with particularly brilliant weather, the sky was overcast, and rain at times seemed imminent.

The company who were present included the Archbishop of Canterbury, the Lord Mayor and Lady Mayoress, Mr. T. W. Wainwright (the treasurer), Dr. Ord, Mr. Mackellar, Mr. Makins (the dean), Mr. Alderman and Sheriff Moore, Mr. Alderman and Sheriff Dimsdale, Alderman Sir James Whitehead, M.P., Sir Joseph Fayrer, K.C.S.I., Sir Edwin Saunders, Sir Spencer Wells, Sir W. MacCormac, Mr. Nettleship, Dr. Semon, and Sir Henry Cuvey (the architect of the new buildings). A small canopy was erected over the platform, which was placed in front of the medical school, and accommodated the Royal party, the Archbishop, and the officiating officers of the hospital. The company were for the most part seated on a platform erected on the grass plot in front, and behind them was the stand for the band of the Coldstream Guards.

Mr. WAINWRIGHT, the Treasurer, spoke as follows:—

It affords me and all officially connected with the hospital great satisfaction that your Royal Highness is able to be present on this important occasion. The interest shown in the progress of this Royal Hospital by her Most Gracious Majesty in laying the first stone, and subsequently opening the new hospital, can never be forgotten, and to-day this large addition to the Medical School buildings is to be declared open by your Royal Highness, whom we rejoice to recognise as our president. It would only weary you were I to attempt to trace the history of this noble charity, founded as it was by an ancestor of your Royal Highness, some 300 years since, in pity for the suffering poor of his day. When the new hospital was designed, very careful estimates were prepared of what the prospective income for hospital purposes was likely to be, and it was fully anticipated that in the course of a few years there would be no uncertainty as to the necessary funds for maintaining the whole hospital for the reception of poor patients, being provided by the estates and funds of the charity, but, unfortunately, this has not been fulfilled—due partly to the unexpected call from the parish authorities for rates, now amounting to £2,500 per annum, and to the sad condition of agriculture; as proof of this I may say that, whereas our rental from country estates in 1872 was £13,000 per annum, and regularly paid, it now only produces £8,000 per annum, and collected with much difficulty. In addition to this reduction in rental, we have in some instances been forced to farm the lands ourselves, which has entailed a very considerable loss, and are obliged to do more in supplying increased buildings and other accommodation in order to keep tenants at all; and beyond all this, under the new Tithe Act, landlords are made liable for the tithe payments, which had previously been regarded as a tenant's payment, but we find practically that it is impossible to add the value of the tithe payments to the rent on the occasion of fresh lettings. Under these circumstances the governors have been unable to meet the increased needs for medical teaching by building those necessary laboratories, lecture rooms, and the like, out of their corporate funds as was originally done, but the medical staff, with true devotion to their important profession, have themselves come forward and provided all that you see before you, making themselves responsible for both interest and capital repayment. I will not enter upon the reason for this large extension, but will leave that to be laid before you by the Dean of the Medical School. I would, however, wish to lay before your Royal Highness the idea that could sufficient funds be provided to pay off the remaining debt upon the hospital, amounting to about £60,000, the governors see their way to open the at present closed wards for reception of poor patients, which would provide for the treatment of 2,000 more in-patients per annum, and when we consider that the building and all appliances are ready, and only the means for maintenance required, I earnestly hope that the liberality of this mighty nation may be trusted to relieve the charity from this debt. When it is also borne in mind that on this side of the river we have only two hospitals—St. Thomas's and Guy's—to serve the population of a district which in 1871 numbered 644,000, and in 1891 had grown to 961,000 (and is still rapidly increasing), the importance of placing St. Thomas's in a position to throw open its whole space for the treatment of the sick poor must be acknowledged by all. And the need is shown by the fact that whilst in 1874 3,502 patients were treated in the wards, in 1893 5,840 were received. I feel that there is one other very important feature in connection with St. Thomas's Hospital, which cannot fail to be deeply interesting to yourself, as holding a high position in the army of this country—namely, that it was after the noble services rendered to our soldiers in the field and hospitals, during the Crimean War, by the band of devoted nurses led by that distinguished and self-sacrificing lady, Florence Nightingale, that the great school of nurses, the first of its kind, was established here, which has been the pioneer of all the improvements made in this important branch of the treatment of the sick both in hospitals and throughout the parishes of the country; and I feel certain, that should the sad occasion again arise, when the services of nurses to tend our sick and wounded soldiers and sailors are called for, those trained under the Nightingale School will be foremost in volunteering for such national work. I can only express a hope that this visit of Your Royal Highness may prove to be the starting point of increased prosperity to the funds of this ancient, Royal, and noble charity, and that before the close of the year we may be enabled to declare the whole hospital, to the full extent of its accommodation, is devoted to the noble work for which it was designed.

Mr. G. H. Makins, the Dean of the Medical School, also read an address, which we hold over until next week, when we hope to publish a plan of the new buildings.

The Duke of Connaught, who was received with loud cheers, said: My Lord, Ladies, and Gentlemen,—I rise to return thanks to Mr. Makins and Mr. Wainwright for the very interesting discourses they have just read us. We stand here, just outside one of the largest and most important of our hospitals—a hospital which shares with Guy's the work on this side of the river. We know how enormously this part of London has extended, and that with this extension the amount of sickness has increased. This hospital—I am afraid it is an old story, but I must repeat it to you to-day—is, like many of our greatest institutions, largely in debt, and it is of the utmost importance that we should try to do all we can to wipe out this burden. (Cheers.) The medical officers have set us a noble example. They have willingly, but at great risk to themselves, embarked in a scheme which will defray the cost of the new building which I am about to open, and we should not be following in their footsteps if we did not try to extinguish the financial obligations of which I have spoken. An ancestor of mine founded it 300 years ago, and at that time he was able to give lands which brought in a certain revenue. We have heard that these lands now bring in only about half the amount, and owing to this, the rates that have to be paid, and other causes the hospital is fettered in the good work it is trying to do. Therefore you I know will forgive me if I appeal most warmly, as President of this hospital, to do all you possibly can to aid an institution which does so admirable a work. I am happy to think that we have with us on this occasion the Chief Magistrate of the City of London. The City and all its great guilds are ever ready to promote what is of benefit to their fellow-creatures, and I trust it is a happy augury that the Lord Mayor has been so kind as to support me on this occasion, and I can assure him that his presence is highly valued by all. (Cheers.) We stand here within the arch of a very fine building, and I must congratulate the architect on its handsome proportions. It must have been a work of some difficulty, with such a huge hospital near, to make the new building harmonise with it, especially when we know that in edifices of this kind architectural beauty has to be combined with utility. We congratulate the architect on the result. (Cheers.) I am glad to think that in connection with this Medical School there has been formed a sort of club for the medical gentlemen attending the hospital. We know the tremendous anxiety and strain of both nerve and brain involved in their work, and recognise that it is necessary they should have some place of relaxation, from which they may return to their duties in the hospital with renewed zeal and energy.

The Archbishop of Canterbury having offered up a prayer. The Duke of Connaught, amid loud cheers, formally declared the building open. A vote of thanks to the Duke was proposed and seconded by Sir Henry Doulton and Dr. Ord. His Royal Highness, after returning thanks, was conducted over the new buildings, and the ceremony concluded.

